# Imported Dengue Case Numbers and Local Climatic Patterns Are Associated with Dengue Virus Transmission in Florida, USA

**DOI:** 10.3390/insects13020163

**Published:** 2022-02-03

**Authors:** Caroline Stephenson, Eric Coker, Samantha Wisely, Song Liang, Rhoel R. Dinglasan, John A. Lednicky

**Affiliations:** 1 Department of Environmental and Global Health, University of Florida, Gainesville, FL 32610, USA; c.stephenson@ufl.edu (C.S.); eric.coker@phhp.ufl.edu (E.C.); songliang@ufl.edu (S.L.); 2 Emerging Pathogens Institute, University of Florida, Gainesville, FL 32610, USA; rdinglasan@epi.ufl.edu; 3 Department of Wildlife Ecology and Conservation, University of Florida, Gainesville, FL 32611, USA; wisely@ufl.edu; 4 Department of Infectious Diseases and Immunology, University of Florida, Gainesville, FL 32608, USA

**Keywords:** dengue virus, transmission, risk, weather, imported, *Aedes aegypti*

## Abstract

**Simple Summary:**

Dengue virus transmission from *Aedes aegypti* mosquitoes to humans is a growing issue in the state of Florida in the United States of America. The majority of cases have occurred in the southernmost counties, even though *Ae. aegypti* are present throughout much of Florida. To understand this geographic partitioning of dengue fever cases in Florida, we compiled ten years (2009–2019) of county-level data on human, environmental, and vector elements and identified risk factors for laboratory-confirmed cases of dengue virus incidence in the state. Counties with the highest average temperatures, highest minimum temperatures, and lowest maximum temperatures were significantly more likely to have local dengue virus transmission. Additionally, moderate rainfall and an increasing number of travel-related dengue cases were also significantly associated with local transmission. This is the first study of its kind to identify county-level risk factors for dengue incidence in Florida. This study provides a parsimonious model that may be useful for prediction of future dengue occurrence based on routinely collected, publicly available data sources. Our findings also highlight the importance of travel-related dengue fever cases to the state as well as environmental conditions that promote dengue virus transmission in Florida by *Ae. aegypti*.

**Abstract:**

*Aedes aegypti* mosquitoes are the main vector of dengue viruses globally and are present throughout much of the state of Florida (FL) in the United States of America. However, local transmission of dengue viruses in FL has mainly occurred in the southernmost counties; specifically Monroe and Miami-Dade counties. To get a better understanding of the ecologic risk factors for dengue fever incidence throughout FL, we collected and analyzed numerous environmental factors that have previously been connected to local dengue cases in disease-endemic regions. We analyzed these factors for each county-year in FL, between 2009–2019, using negative binomial regression. Monthly minimum temperature of 17.5–20.8 °C, an average temperature of 26.1–26.7 °C, a maximum temperature of 33.6–34.7 °C, rainfall between 11.4–12.7 cm, and increasing numbers of imported dengue cases were associated with the highest risk of dengue incidence per county-year. To our knowledge, we have developed the first predictive model for dengue fever incidence in FL counties and our findings provide critical information about weather conditions that could increase the risk for dengue outbreaks as well as the important contribution of imported dengue cases to local establishment of the virus in *Ae. aegypti* populations.

## 1. Introduction

The geographic distribution of the principal dengue virus (DENV) vector *Aedes aegypti (Ae. aegypti)* is predicted to cover much of the southeastern United States of America (USA) over the next thirty years [1]. There are DENV serotypes: DENV-1, -2, -3, and -4. These viruses are the causative agents of dengue fever (“dengue”), the most prevalent arthropod-borne virus illness in the world [2]. Globally, dengue is responsible for an immense human health and economic burden [3]. Nearly half of the world’s population lives in at-risk regions for dengue, and those regions are expected to expand in coming years [1,4]. There are over 50 million symptomatic dengue cases yearly, with many more predicted to be asymptomatic, and nearly 20% of dengue cases require hospitalization [5]. Dengue also results in a global cost of almost USD 9 billion [5]. It is important to understand the nuances of transmission in areas where dengue has the possibility to become endemic so as to halt the increasing burden to humans and the global economy.

The expanding range of these mosquito vectors is predicted to also increase risk for autochthonous (local) dengue transmission into new areas. Thus far, Texas and Florida (FL) have been the sole states from the contiguous USA that have reported almost yearly local dengue transmission [6]. Florida’s economy is largely dependent on tourism which could be a contributing factor to the number of travel-related introductions of DENV to the state [7]. For example, in 2019, there were 397 travel-associated DENV cases [8]. Currently, the factors that influence local dengue transmission in FL are poorly understood. Although studies that considered personal-level risk factors for dengue virus exposure have been conducted in FL and TX previously [9,10], more work is needed to predict where dengue occurs in these states to better inform local vector and disease control programs. Additionally, a better understanding of the factors associated with the spread of dengue into non-endemic regions in general can be useful to limit the growing reach of this disease globally.

*Ae. aegypti* are present throughout most of peninsular FL but are absent from the panhandle [11]. Many FL counties are found to have *Ae. aegypti* in addition to the secondary dengue vector, *Aedes albopictus* [11]. Though presence of the principal or secondary vector is a prerequisite for local DENV transmission, not every county in FL with *Ae. aegypti* has reported local transmission over the last twelve years. Nearly all autochthonous transmission of DENV in FL has occurred in the southern half of the state with the majority of cases in the southernmost counties [6]. For this study, the state of FL served as the study area due to its ideal *Ae. aegypti* environment, proximity to DENV endemic areas in the Caribbean, Central and South America, and the increasing number of locally acquired cases in the state in recent years. Despite having several vector control programs in the state, resources to support this effort are not evenly allocated throughout FL. The goal of these analyses was to develop a predictive model of dengue case occurrence in FL counties using factors related to humans, environmental and the vector, which can inform the allocation of resources towards more targeted control activities. Here, we hypothesize that county-level average temperature and precipitation are significant predictors of county-level dengue occurrence in FL.

Human population density is an important factor to consider when modeling dengue occurrence because *Ae. aegypti* are a highly anthropophilic species that is almost universally found in areas of high human population density [12]. DENV is not currently thought to be endemic across FL, so transmission is dependent on local introductions by travelers or the persistence of infections in the mosquito populations following a local outbreak. As such, it is crucial to also account for imported cases to the state to better predict potential DENV transmission [13,14,15]. Importation of arboviruses is not only a prospective issue to regions of the USA but is also an ongoing international problem, especially in Europe [16,17,18]. For example, over 15% of travel-related febrile illness is caused by dengue [19]. Florida is a tourism hub and a hotspot for international travel. It would be ideal to minimize local dengue transmission in FL as much as possible to also prevent any travel-related infections exported out of the state. In the Americas, morbidity and mortality from dengue has been worsening in recent decades [20]. Preventing dengue from taking a foothold in the USA would also limit the increase of regional transmission cycles in the Americas.

Regarding climate variables, temperature, precipitation and humidity are all factors that affect a mosquito’s extrinsic incubation period for viruses (the time from virus uptake by the vector to transmission to a human host), feeding rates, and survival [21]. In a recent meta-analysis, both temperature and precipitation were significantly associated with dengue fever risk, primarily in Southeast Asia and several studies from Brazil. [22]. Average temperature, minimum temperature, and wind speed were also found to strongly correlate with dengue incidence in Colombia [23], and rainfall and maximum temperature were related to dengue prevalence in Pakistan [24]. The built-environment is a well-established contributing factor to vector presence and abundance globally. For instance, roadways in FL have been linked to genetic diversity among *Ae. aegypti* populations [25] by acting as barriers between populations as well as a means to transport mosquitoes outside of their normal flight range. The urban built-up environment has been positively associated with dengue in Thailand and Taiwan [26,27]. *Ae. aegypti* is an urbanized vector so dengue transmission in FL may also follow a similar trend of occurring in counties with the highest percent urbanized land cover.

Increasing our knowledge of the risk factors for dengue transmission, especially in non-endemic areas, can aid prevention by flagging potential hotspots in hopes of mobilizing control efforts to those areas. Herein we used a negative binomial regression model, incorporating a wide range of predictors across the human, environmental and vector dimensions, to understand drivers of dengue virus transmission in FL.

## 2. Materials and Methods

### 2.1. Study Area and Risk Factor Data Collection

Forty counties in FL across eleven years (2009 to 2019) were included in the analysis, reflecting 440 county-years. Counties from the panhandle (*n* = 27) were excluded since *Ae. aegypti* is not present, and therefore, dengue transmission is not likely to occur in these counties. Data were gathered on potential risk factors for dengue transmission in FL from several publicly available sources (Table 1). Mapping and spatial analyses were performed using ArcGIS (ESRI 2019, ArcGIS Desktop: Release 10.7.1. Redlands, CA: Environmental Systems Research Institute). Statistical analyses were conducted using SPSS statistical software (IBM Corp. Released 2020. IBM SPSS Statistics for Windows, Version 27.0. Armonk, NY, USA: IBM Corp).

### 2.2. Response Variable

The response, or dependent, variable was number of local human dengue cases per county-year. This variable ranged from 0 to 66 cases and had an average of 0.36 cases per county-year. Data were shared by the Florida Department of Health for confirmed and probable cases reported to the Florida Department of Health, classified using the Council of State and Territorial Epidemiologists national surveillance case definition [28].

### 2.3. Predictor Variables

#### 2.3.1. Human

Imported dengue case numbers by month and year per county were shared by the Florida Department of Health. Imported/travel cases ranged from 0–234 for any given county per year. Data were obtained from the American Community Survey (ACS) from the U.S. Census Bureau (census.gov) to compute population density (people per square mile) per county-year. Population density ranged from 15.69–3577.64 people/square mile.

#### 2.3.2. Weather

Data were extracted from the Florida Automated Weather Network (FAWN) for monthly averages of maximum, average and minimum temperature per year (converted from degrees Fahrenheit to degrees Celsius (°C)), humidity (%), rainfall (converted from inches to centimeters (cm)) and wind speed (converted from miles per hour to kilometers per hour (km/h)) (fawn.ifas.ufl.edu). FAWN provides monthly averages of each variable from all forty weather stations in their network. As dengue transmission has been most prominent between the months of May through October over the last eleven years, we calculated average weather variables across those six months per county. Data during months in which a hurricane or tropical storm occurred in FL was removed, to avoid biased measurements due to extreme weather events. Weather stations are not located in every county, so we used ArcGIS to interpolate weather values at unmonitored counties using point data from each weather station across the state using inverse distance weighting (IDW). IDW is commonly used for weather-related factors because it assumes spatial autocorrelation where areas that are closer together have similar values. The weights in IDW are proportional to the inverse distance between the point and its predicted location. After interpolation, we were able to assign a weather value for each of the 40 counties included in the analysis for 2009 through 2019.

Average temperature ranged from 23–27 °C and was reclassified into quartiles. Quartiles were then included as a categorical (factor) variable into the model. We selected quartiles in the model by comparing Akaike information criterion (AIC) between models that fit the weather variables as the categorical quartiles versus (vs.) continuous predictor values (AIC: 584 vs. 745, respectively). The AIC is a relative measure of model fit between iterations, and lower values indicate a better fit of the model. Maximum temperature ranged from 33.3–38.3 °C and the categorized data by quartiles had a better model fit (AIC: 435) versus the continuous values (526). Likewise, minimum temperature (range: 7.7–20.8 °C, AIC: 661 vs. 678), rain (range: 6.35–25.4 cm, AIC: 625 vs. 788) and humidity (range: 76–86%, AIC: 575 vs. 794) were all fit as quartiles. Wind ranged from 2–6 mph and performed best in the model as a continuous measure (AIC: 655 for categorical, 623 for scale). We also included a variable for number of days experiencing a tropical storm or hurricane for each county-year, referred to as “hurricane days”, obtained from reports from the National Oceanic and Atmospheric Administration (NOAA) (noaa.gov); this variable ranged from 0–7 days.

#### 2.3.3. Built-Environment

MODIS MCD12Q1 remotely sensed land cover data (MODIS Land Cover V6) were extracted from the U.S. Geological Survey (USGS) (usgs.gov) [29]. We used land cover data for each year between 2009 through 2019. There were 19 different land cover types, of which we were only interested in the “urban and built-up” variable. An area is designated as urban and built up by the MODIS algorithm if 30% or greater of the land has impervious surface area due to buildings, asphalt or vehicles [30]. We calculated the sum of the area for each county and then subtracted land area designated as permanent wetlands or water since these are uninhabitable and could confound county-wide measures of percent urban/built-up environment of habitable lands. Our final variable was percent urban/built-up landscape out of the total county area minus the area of permanent wetlands and water, which we refer to as “percent urban” throughout. This variable was included as a quantitative variable in further statistical modeling and ranged from 0.045–84.6%.

Data on km of highways per county between 2009 and 2019 were obtained from the Florida Department of Transportation (fdot.gov). This measure included interstates, highways, and freeways across each county, which are high speed roadways, ranging from zero to 300.8 km. Kilometers of highways were included as a quantitative variable in the model.

#### 2.3.4. Vector

We received records for mosquito surveillance across FL dating from 2009 through 2019 that were collected by various Mosquito Control Districts across FL and compiled and shared by the Cummings Laboratory at the University of Florida. Across those years, FL counties had variable number of trapping days, number of trap sites and trap types deployed. The variability between counties made it difficult to compare baseline mosquito abundance of *Ae. aegypti* and *Ae. albopictus,* as originally intended. After calculating yearly estimates of both *Ae. aegypti* and *Ae. albopictus* per county, we had 30% coverage across the forty study counties between 2009–2019 and 70% missing values. This corresponded to data on 132 county-years out of the total 440 county-years. Across the records, there were on average 4.5 county years of data for each county between 200–2019. We calculated a standardized measure of the ratio between *Ae. aegypti* to *Ae. albopictus* per county year to be able to make comparisons and inferences about the relative abundance of the principal DENV vector to the secondary vector between counties. The ratio of *Ae. aegypti* to *Ae. albopictus* ranged from 0 to 890, the average county-year had a ratio of 11.66 and a median ratio of 0.45.

### 2.4. Spatial Autocorrelation 

The presence of spatial autocorrelation, the degree to which an object is similar to its neighbors, was assessed using Moran’s I [31]. Moran’s I is an inferential statistic and variant of the correlation coefficient. In this study, we defined counties to be “nearby” to another county if they share a border or vertex with another county.

Using the calculated value of Moran’s I we then calculated a standardized Z value to determine significance of the measured spatial autocorrelation. Z values that are greater than 1.96 or less than −1.96 indicate a rejection of the null hypothesis, that there is random dispersion. Z values between 1.96 and −1.96 indicate that the null hypothesis cannot be rejected, and spatial randomness is assumed. We tested for spatial autocorrelation on the response variable, number of local dengue cases per county-year, as well as the residuals of the final model based on the training dataset.

### 2.5. Model Building Using Negative Binomial Regression 

We partitioned the 440 county-year database into a training and testing dataset by assigning random numbers to each of the records and creating a subset with the 20% lowest random numbers (*n* = 88) for the testing data, and leaving the remaining 80% (*n* = 352) records to build and train the statistical model to predict the occurrence of dengue in FL. Our outcome of interest was case counts of dengue in FL between 2009–2019. Poisson regression and negative binomial regression are common statistical methods to analyze count data (positive integers), especially for low occurrence outcomes. Poisson regression has a strict assumption of equidispersion, meaning that the mean and variance are equal. Negative binomial regression does not have as strict an assumption about equidispersion and can be used with overdispersed data, where the variance is greater than the mean. Upon testing the assumption of equidispersion, it was found that the data did have overdispersion (mean: 0.39, variance: 15.4). We therefore built and validated a statistical model to predict dengue occurrence using negative binomial regression. In addition to a count outcome, negative binomial regression must also have at least one or more predictor variables that are categorical (nominal or ordinal) or continuous. When the response variable is a count outcome with variable exposure among the population(s) of interest, an offset variable can be used to compensate for this. The model herein included the natural log of population size per county [ln(population size)] as the offset variable since counties with more people overall will have a larger pool of dengue-susceptible people. 

We evaluated univariate associations of each variable with the outcome and then included all significant factors into multivariable model iterations. Overall model significance was assessed by the omnibus test, and the test of model effects was used to determine significance of each parameter based on Wald Chi-Square test *p*-value. Significance tests were performed at an α level of 0.05 and variables were dropped from the multivariable model if the Wald Chi-Square test *p*-value was not significant. If a model passed all significance tests, the AIC was then evaluated and the model of best fit selected based on the lowest AIC. The output of binomial regression is the incident rate ratio (IRR), which is the exponentiated beta coefficient (β). An IRR is a relative measure to compare incidences between two events, otherwise considered to be an exponential effect size measure [32]. We also calculated a secondary measure of effect size known as the standardized mean difference (SMD) effect size, which is the difference between two group means divided by the standard deviation and can be calculated using a web interface designed by Coxe, 2018 [32].

### 2.6. Model Validation

After determining the model of best fit by comparing AICs between models in the training set, each parameter estimate was included into the full additive equation that incorporates the linear combination of predictors equating to the log of the outcome using the 20% held out test set:

(1) $$log\left(dengue\;cases\right)\;=\;Intercept+\beta 1+\beta 2+\beta 3\ldots \beta n$$

The equation above, where β_1_ (beta coefficient one) is the regression coefficient for variable one added to each subsequent beta coefficient through variable *n,* was then applied to the testing dataset to predict case counts per county year. We predetermined that only those outcomes of positive integers would be counted as “cases” and that the predicted outcome would be categorized into groups of “1 = predicted presence (case counts < 1)” or “0 = predicted absence (case counts < 1)”. We then calculated model sensitivity, specificity, accuracy, where *a* is both predicted presence (by the model) and observed presence (actual historical health department records) of local dengue cases, *b* is predicted absence of dengue cases but observed presence, *c* is predicted presence but observed absence and *d* is predicted absence and observed absence.

(2) $$sensitivity=\frac{a}{\left(a+b\right)}*100,$$

(3) $$specificty=\frac{d}{\left(c+d\right)}*100,$$

(4) $$accuracy=\frac{a+d}{a+b+c+d}*100,$$

and the kappa statistic (*K*) to remove chance agreement from accuracy measures

(5) $$chance\;agreement=\left(\frac{a+b}{a+b+c+d}*\frac{a+c}{a+b+c+d}\right)+\left(\;\frac{b+c}{a+b+c+d}*\frac{c+d}{a+b+c+d}\right),$$

(6) $$K=\frac{accuracy-chance\;agreement}{\left(1-change\;agreement\right)},$$

using an error matrix that displays “*a*” as true positives, “*b*” as false negatives, “*c*” as false positives and “*d*” as true negatives (Table 2).

## 3. Results

### 3.1. Univariate Associations between Predictors and Dengue Cases

In the training dataset, population density was not significantly associated with dengue case counts. Population density also exhibited multicollinearity with other predictors in the model and was thus not included in the full model. When the model included the *Ae. aegypti*:*Ae. albopictus* ratio per county, 72% of records were dropped due to missing data. This variable was thus excluded from model building since this variable was not missing at random. All weather-related factors (average temperature, maximum temperature, minimum temperature, rain, wind, and humidity) were significantly associated with dengue cases except for hurricane days. Additionally, imported case numbers, km of roads per county and percent urban were also associated with dengue case counts in bivariate models.

### 3.2. Model Training

All variables that were independently and significantly associated with dengue case counts were included in the full model for training. The full model had a significant omnibus test (*p* < 0.001) and an AIC of 218.665, but wind speed (*p* = 0.081) and km of highways (*p* = 0.163) were no longer significant and were removed from the next iteration. After removing wind speed and km of highways, the overall model fit was significant (*p* < 0.001), but the AIC increased to 220.368 and humidity was no longer significant (*p* = 0.41). Taking out humidity resulted in a significant model overall and every predictor was also significant via Wald Chi-Square test. However, the AIC further increased to 222.29. We were interested to know if there was an interaction occurring between percent urban and maximum temperature and included that term into the model. Including the interaction term between percent urban and maximum temperature decreased the AIC to 217.378, the omnibus test was still significant (*p* < 0.001). Our final and best fitting model (Table 3) had an AIC of 212.14, a significant omnibus test (*p* < 0.001), and all predictors were significantly associated with dengue case counts, including average temperature, maximum temperature, minimum temperature, rainfall, imported dengue case numbers, and the interaction between maximum temperature and percent urban.

**Figure 1:**
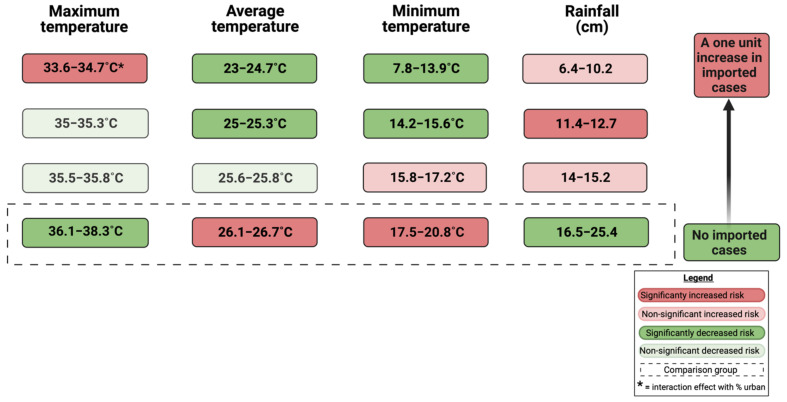
Conceptual model of the risk for dengue occurrence in Florida, USA.

A conceptual model for the statistical model of best fit is presented in Figure 1. Counties with an average temperature between 23–24.7 °C (based on temperature quartiles) had a dengue incident rate ratio 0.058 times that of counties with an average temperature between 26.1–26.7 °C (*p* < 0.001) (Table 3). Similarly, counties with an average temperature between 25–25.3 °C had a dengue incident rate 0.026 times that of counties with an average temperature between 26.1–26.7 °C (*p* < 0.001). Lastly, counties with an average temperature between 25.6–25.8 °C had a dengue incident rate 0.122 times that of counties with an average temperature between 26.1–26.7 °C (*p* = 0.009). In summary, an increase of average temperature is (non-linearly) associated with increasing dengue case numbers. Counties with a maximum temperature between 33.6–34.7 °C had a dengue incident rate 46.418 times that of counties with a maximum temperature between 36.1–38.3 °C (*p* = 0.041). The other comparisons were not statistically significant. Counties with 11.4–12.7 cm of monthly rain had a dengue incident rate 6.803 times that of counties with 16.5–25.4 cm of rain (*p* = 0.009). Counties with a minimum temperature between 7.8–13.9 °C had a dengue incident rate 0.093 times that of counties with a minimum temperature between 17.5–20.8 °C (*p* = 0.039). Counties with a minimum temperature between 14.2–15.6 °C had a dengue incident rate 0.025 times that of counties with a minimum temperature between 17.5–20.8 °C (*p* = 0.008). Thus, an elevation of minimum temperature was associated with increased dengue incidence. A one unit increase in number of imported dengue cases corresponded to a 1.091% increase in the rate of local dengue cases (*p* < 0.001). Finally, the interaction between percent urban and lowest maximum temperature of 33.6–34.7 °C indicated that when there was a one unit increase in percent urban, the effect of low maximum temperature decreases by 16.8%. An increase in the urban environment can decrease the magnitude of risk that a milder maximum temperature has on dengue incidence.

### 3.3. Spatial Autocorrelation

Neither the dependent variable nor the residuals of the model had significant spatial autocorrelation. The Moran’s I value of the dependent variable was 0.01, the Z value was 0.47, and the *p*-value was 0.68. The Moran’s I value of the residuals was 0.006, the Z value was 0.30 and the *p*-value was 0.63. Both values were slightly positive, indicating a minimal tendency toward clustering, but *p*-values corresponding to each Z value were not statistically significant. It can be concluded that there is no evidence in our data for residual spatial confounding due to spatial autocorrelation in the model.

### 3.4. Model Testing and Validation

We applied the additive equation derived from the model of best fit to the testing dataset to predict case counts. Precited outcomes were dichotomized, and any predicted positive integer was coded as “1” = any number of predicted cases, and all other instances were coded as “0” = no predicted cases. The model predicted 59 dengue cases in 2019 when there were truly 16 cases that year. The model did not predict cases in any other county within the testing dataset, although there was one case in Martin Co., FL, USA in 2011 and seven cases in Miami-Dade Co., FL, USA in 2014. Per the pre-determined case dichotomization, the outcomes were reported as one instance of correctly predicted dengue and two instances of missed occurrences of dengue. The model correctly predicted the remaining 85 instances of dengue absence. We report these predictions as 85 instances of correctly predicted absences with no occurrences of incorrectly predicted absences. The predictive model had 97.7% accuracy and a kappa statistic of 0.976 which corresponds to almost perfect agreement. The sensitivity was 33% and the specificity was 100%. The largest effect sizes for this model were for the lowest quartile of maximum temperature (IRR: 46.418 and SMD: 0.071) and for moderate rainfall (IRR: 6.803 and SMD: 0.009) are classified in the small effect range.

## 4. Discussion

We identified human and environmental predictors for dengue incidence in FL. As expected, climate-related factors appear to be most impactful since average temperature, minimum temperature, maximum temperature, and rainfall were all significant predictors of dengue occurrence in FL. Number of imported cases was also associated with dengue occurrence in the state, as was the interplay between degree of urban development and maximum temperature. In summary, people in counties with the highest average temperature, most mild maximum temperature, most mild minimum temperature with mid-range rainfall (11.4–12.7 cm) have the highest risk for dengue infection. Additionally, people in counties with elevated imported dengue cases are also at a higher risk for dengue. Our validated model may be of use in other sub-tropical and temperate areas where dengue is non-endemic but has the potential to propagate through autochthonous cases. In such countries internationally, epidemiologic [17], environmental [33], and entomological [34] surveillance have been found to be useful tools for understanding dengue outbreaks. Our work brought in data across these different dimensions to understand the risk factors for dengue transmission and could be a useful tool to identify potential risk areas for dengue in similar regions if validated in that location.

It is not surprising that climate-related factors were highly associated with dengue incidence in FL. Globally, temperature, humidity, rainfall, and wind have all been identified as risk factors for dengue elsewhere due to their connection to mosquito abundance [22,23,35]. However, the results from the present study highlight certain temperature and rainfall ranges in which risk for dengue occurrence is significantly increased. *Ae. aegypti* survival is impaired at extreme temperatures [36] with developmental impacts below 14.5 °C and above 32 °C [37], which would explain why counties with a higher average temperature, within that range, yet fewer extreme temperatures, outside of that range, would have the highest risk for dengue. This would be particularly important protective factor for areas that can reach extreme highs during the day time since *Ae. aegypti* are primarily daytime biters, after sunrise and before sunset [38]. Similarly, rain is necessary to fill outdoor containers that provide habitats for *Ae. aegypti* eggs to hatch and larvae and pupae to mature, as *Ae. aegypti* are known to commonly use these types of containers to oviposit in FL [39] and elsewhere [40,41]. Too much rainfall, however, could cause a flushing effect that reduces vector abundance [42].

Although lower maximum temperatures are a substantial risk factor for dengue incidence in this model (IRR: 46.418), compared to more elevated maximum temperatures, the interaction of this variable with percent urban landcover suggests that highly urban areas can have a high risk of dengue transmission even at the most extreme temperatures. It was intriguing that percent urban alone was not significantly associated with dengue incidence, but its interaction with maximum temperature lessens the impact of higher temperatures. This interaction could be due to Urban Heat Islands (UHIs), where heat is generated by the built environment when it consumes and re-radiates solar radiation. [43] UHIs and microclimates have been associated with increased dengue incidence previously [44,45]. Therefore, it could be that a milder maximum temperature is more of a risk factor for dengue transmission outside of highly urban areas. In the future we aim to conduct analyses at higher resolution within dengue hotspots, like Miami-Dade or Monroe counties to understand the impact of microclimates in urban settings on dengue incidence within hotspot communities.

Our results provide an important foundation for understanding the dynamics of DENV transmission in FL. This is the first study of its kind to develop and validate a predictive model for dengue incidence in the state. This model has high accuracy (97.7%) and specificity (100%), but less than desirable sensitivity (33%). We are confident in this model’s ability to predict low and high dengue risk areas, but one limitation of the model is that it may underperform when detecting very low case numbers. The model accurately identified dengue incidence in the county with the highest case numbers in the testing dataset (*n* = 16 for Miami-Dade in 2019) but failed to detect dengue incidence in two other counties with lower incidence (*n* = 1 & *n* = 7). The low sensitivity may be a result of the overall low incidence of dengue in FL, but the model could be refined further in the future. The model could be adapted to a machine learning method. Machine learning algorithms can accommodate missing data better than statistical models [46]. We believe that *Ae. aegypti*:*Ae. albopictus* ratios would have been an important factor in the model if there was more coverage over county-years due to a noticeably higher ratio of *Ae. aegypti* in counties with previous dengue transmission in our current database. A machine learning approach could enable us to incorporate these data and such algorithms have been found to outperform regression and timeseries analyses [47,48]. Additionally, though we verified that there was no spatial autocorrelation in these data, a limitation of this work is that we did not analyze temporal autocorrelation. In the future, we aim to better understand potential temporal trends occurring year by year. Additionally, though many predictors in the model are statistically significant the overall effect sizes are in the small effect range, indicating that other factors could also be contributing to dengue occurrence in FL. We could explore additional factors in the human, environmental and vector dimensions in a future analysis. A final limitation of this work is that the model, though useful locally in FL, USA, is not validated for use in other regions or nations. For use outside of our study location, this model will need to be validated against ground truth data in other locales. Nonetheless, our work still provides the first insights into county-level risk factors for dengue transmission in FL. We envision that this model can provide a framework for predicting dengue incidence throughout the state. 

Statistical models can be useful tools for predicting areas of suitability for *Ae. aegypti* as well as predicting disease incidence [27,49,50,51,52]. Poisson and negative binomial regression have been commonly used to model dengue incidence, and many studies globally have uncovered that weather-related factors such as rainfall, temperature and humidity in a given location can be useful predictors of dengue [53,54,55]. Much remains to be investigated regarding personal, community, and state-wide risk factors for DENV transmission in FL, but this work contributes to a better understanding of several risk factors related to climate, the built-environment and imported dengue case numbers. Previous work revealed several individual-level risk factors for dengue in FL, including bird baths and vegetation around residences as well as open windows [9,56] and our results contribute additional evidence that at the county-level, higher average temperatures, moderate rainfall, and fewer extreme temperatures coupled with increasing urban population and an increasing number of imported cases could result in conditions favorable for DENV transmission in FL. Our work overall is in line with the need for a more wholistic approach to arbovirus prevention and control that considers the wider dimensions of human health, vector biology and environmental science [57,58]. Finally, these findings contribute to potential wider reaching implications to support the prevention of autochthonous cases wherein the virus could become established in the local mosquito population or have the potential to spread as travel cases to other nations.

## Figures and Tables

**Table 1:** Human, environmental and vector-related factors by county-year included in predictive model building for dengue occurrence in Florida, USA.

Level/Predictor	Units	Source
Human		
Population density	People per square kilometer	U.S. Census Bureau American Community Survey
Imported dengue	Number of cases	Florida Department of Health via Dr. Andrea Morrison
Environment		
Average temperature	Degrees Celsius (°C)	Florida Automated Weather Network (FAWN)
Maximum temperature	Degrees Celsius (°C)	FAWN
Minimum temperature	Degrees Celsius (°C)	FAWN
Humidity	Percent	FAWN
Rainfall	Centimeters (cm)	FAWN
Wind speed	Kilometer per hour (km/h)	FAWN
		
Urban/built-up	Percent total county area (minus wetlands and water)	United States Geological Survey (USGS) MODIS Land Cover Version 6
Highway kilometers	Kilometers	Florida Department of Transportation (FDOT)
Hurricane days	Number of days experiencing hurricanes or tropical storms per year	National Oceanic and Atmospheric Administration (NOAA)
Vector		
*Aedes aegypti*:*Aedes albopictus* abundance	Relative ratio	Dr. Derek Cummings, Florida Mosquito Control Districts, Florida Department of Agriculture and Consumer Services

**Table 2:** Error matrix of predicted and observed dengue presence and absence for model validation.

	Predicted Presence	Predicted Absence
Observed presence	1 (*a*)	2 (*b*)
Observed absence	0 (*c*)	85 (*d*)

**Table 3:** Test of model effects and parameter estimates for final selected predictive model for dengue incidence in Florida, USA.

Source	Parameter Estimate (β)(95% Confidence Interval [CI])	*p*-Value	IRR	Standard Error	Standardized Mean Difference Effect Size
(Intercept)	−12.928 (−16.7, −9.1)	0.000	< 0.001	1.9291	--
Average temperature (°C)	--	<0.001	--	--	--
23–24.7	−2.842 (−4.4, −1.3)	<0.001	0.058	0.8097	−0.001 ^+^
25–25.3	−3.650 (−5.4, −1.9)	<0.001	0.026	0.8975	−0.002 ^+^
25.6–25.8	−2.107 (−3.7, −0.5)	0.009	0.122	0.8109	−0.001 ^+^
26.1–26.7 *	--	--	1	--	
Maximum temperature (°C)	--	<0.001	--	--	--
33.6–34.7	3.838 (0.2, 7.5)	0.041	46.418	1.8792	0.071
35–35.3	−2.299 (−6.6, 2.0)	0.295	0.100	2.1944	−0.001
35.5–35.8	−4.,772 (−9.7, 0.2)	0.062	0.008	2.5599	−0.002
36.1–38.3 *	--	--	1		
Minimum temperature (°C)	--	0.009	--	--	--
7.8–13.9	−2.375 (−4.6, −0.1)	0.039	0.093	1.1481	−0.001 ^+^
14.2–15.6	−3.700 (−6.4, −1.0)	0.008	0.025	1.3872	−0.002 ^+^
15.8–17.2	0.683 (−0.8, 2.1)	0.353	1.981	0.7353	0.002
17.5–20.8 *	--	--	1	--	--
Rain (cm)	--	<0.001	--	--	--
6.4–10.2	1.070 (−1.3, 3.5)	0.382	2.916	1.2247	0.003
11.4–12.7	1.917 (0.5, 3.3)	0.009	6.803	0.7286	0.009 ^+^
14–15.2	0.469 (−1.2, 2.2)	0.592	1.598	0.8734	0.001
16.5–25.4 *	--	--	1	--	--
Imported dengue cases	0.087 (0.04, 0.14)	<0.001	1.091	0.0257	0.0001 ^+^
Maximum temperature (°C) * Percent urban %	--	<0.001	--	--	--
33.6–34.7 °C x % urban	−0.184 (−0.2, −0.1)	<0.001	0.832	0.0258	−0.0002 ^+^
35–35.3 °C x % urban	−0.081 (−0.2, 0.02)	0.119	0.922	0.0520	0
35.5–35.8 °C x % urban	0.007 (−0.1, 0.1)	0.899	1.007	0.0583	0
36.1–38.3 °C x % urban	−1.035 (−2.9, 0.8)	0.279	0.355	0.9560	−0.001

* Denotes comparison groups and “x” denotes an interaction; ^+^ Denotes statistical significance.

## Data Availability

Data available upon request.
